# Impact of idiopathic pulmonary fibrosis on advanced non-small cell lung cancer survival

**DOI:** 10.1007/s00432-016-2199-z

**Published:** 2016-06-27

**Authors:** Nobuhiro Kanaji, Akira Tadokoro, Nobuyuki Kita, Makiko Murota, Tomoya Ishii, Takehiro Takagi, Naoki Watanabe, Yasunori Tojo, Shingo Harada, Yusuke Hasui, Norimitsu Kadowaki, Shuji Bandoh

**Affiliations:** 1 Department of Internal Medicine, Division of Hematology, Rheumatology and Respiratory Medicine, Faculty of Medicine, Kagawa University, 1750-1 Ikenobe, Miki-cho, Kita-gun, Kagawa, 761-0793 Japan; 2 Department of Radiology, Faculty of Medicine, Kagawa University, Kagawa, Japan; 3 Department of Respiratory Medicine, National Hospital Organization Takamatsu Medical Hospital, Kagawa, Japan; 4 Department of Internal Medicine, Takamatsu Heiwa Hospital, Kagawa, Japan; 5 Department of Cardiovascular Medicine, Kagawa Prefectural Shirotori Hospital, Kagawa, Japan

**Keywords:** Interstitial lung disease, Chemotherapy, Docetaxel, Acute exacerbation, Epidermal growth factor receptor, Advanced lung cancer

## Abstract

**Purpose:**

The clinical features of patients with advanced non-small cell lung cancer (NSCLC) and interstitial lung disease (ILD) have not fully been elucidated. This study aimed to investigate the clinical features of these patients, particularly with idiopathic pulmonary fibrosis (IPF).

**Methods:**

Data on 218 patients with pathologically confirmed diagnoses of NSCLC who had been treated with chemotherapy and/or molecular targeted therapy were retrospectively analyzed for progression-free survival (PFS), overall survival (OS), responses to first-line therapy, and incidence of acute exacerbations (AEs).

**Results:**

Fifty-three of the 218 patients were diagnosed with ILD, and 34 of them with IPF. The frequency of epidermal growth factor receptor (*EGFR*) mutation was significantly lower in ILD and IPF patients than in non-ILD patients (2 or 0 vs. 32 %, respectively). Median PFS and OS were significantly shorter in both ILD and IPF patients than in non-ILD patients (118, 92, and 196 days for PFS, and 267, 223, and 539 days for OS, respectively). Multivariate analysis showed that poor performance status, absence of *EGFR* mutation, and presence of IPF were poor prognostic factors for PFS and OS. Disease control rate (DCR) was significantly lower in ILD and IPF patients than in non-ILD patients regardless of the presence of *EGFR* mutation (67 or 53 vs. 85 %, respectively). The incidence of AEs of ILD was significantly higher during chemotherapy with docetaxel-containing regimens (seven of 38; 18.4 %).

**Conclusions:**

Both IPF and ILD were associated with lower *EGFR* positivity, lower DCR, and shorter PFS and OS in advanced NSCLC patients.

## Introduction

Interstitial lung diseases (ILDs), also called interstitial lung abnormalities (ILA) or interstitial pneumonia (IP), are characterized by diffuse pulmonary interstitial abnormalities that often lead to fibrosis (Borchers et al. 2011). Recent evidence has shown that preexisting ILD is associated with shorter survival in patients with advanced non-small cell lung cancer (NSCLC) (Kinoshita et al. 2012; Nishino et al. 2015). In a study of NSCLC patients receiving chemotherapy, 22 of whom had idiopathic interstitial pneumonia (IIP), IIP was found to be a significantly unfavorable factor for progression-free survival (PFS) (95.0 vs. 199.5 days) and overall survival (OS) (163.0 vs. 400.0 days) (Kinoshita et al. 2012). In another study, NSCLC patients with high ILA scores according to computed tomography (CT) findings had shorter OS (*n* = 17, 7.2 months) than those without ILA (*n* = 103, 14.8 months) (Nishino et al. 2015).

Idiopathic pulmonary fibrosis (IPF), the commonest type of ILD (Borchers et al. 2011), is defined as a specific form of chronic, progressive fibrosing IP of unknown cause, occurring primarily in older adults, limited to the lungs, and associated with the histopathologic and/or radiologic patterns of usual interstitial pneumonia (UIP) (Raghu et al. 2011). IPF was recently reported to be a poor prognostic factor in patients with surgically treated NSCLC (Goto et al. 2014; Lee et al. 2014). Only one study has focused on the efficacy of chemotherapy in advanced NSCLC with IPF (Watanabe et al. 2013). The authors reported that 21 patients with IPF had an overall response rate (RR), PFS, and OS of 42.9 %, 5.4, and 11.4 months, respectively (Watanabe et al. 2013). No published studies have compared the survival of IPF and non-ILD patients with advanced NSCLC.

Because there is limited clinical information regarding advanced NSCLC patients with IPF, we retrospectively compared the efficacy of chemotherapy and survival in NSCLC patients with ILD or IPF with that in patients without ILD. In addition, we investigated the incidence, risk factors, and outcome of acute exacerbations (AEs) after receiving chemotherapy.

## Materials and methods

### Patients

This study was approved by the Institutional Review Board of Kagawa University. Patients with pathologically confirmed advanced (stage IIIB or IV) NSCLC who presented to the Department of Internal Medicine, Kagawa University Hospital, between January 2007 and July 2015 were retrospectively identified, and relevant clinical and laboratory data were collected from their medical records. Patients who (1) had received definitive thoracic irradiation (usually more than 60 Gy), (2) had another concomitant active malignancy, or (3) had received only best supportive care were excluded, whereas those treated with chemotherapy and/or molecular targeted therapy such as tyrosine kinase inhibitors (TKIs) for epidermal growth factor receptor (EGFR) were included.

### Classification of ILD and diagnosis of IPF and AE

The classification of ILD was made in accordance with the ATS/ERS/Japanese Respiratory Society/Latin American Thoracic Association Statement (Raghu et al. 2011). UIP pattern was defined as having all the following four features: (1) subpleural, basal predominance; (2) reticular abnormality; (3) honeycombing with or without traction bronchiectasis; and (4) absence of features listed as inconsistent with UIP pattern (Raghu et al. 2011). If honeycombing was absent, but other features met the criteria for UIP, the case was classified as possible UIP pattern (Raghu et al. 2011).

The diagnosis of IPF was based on the following criteria: (1) UIP pattern on high-resolution computed tomography and (2) exclusion of other known causes of interstitial lung diseases (Raghu et al. 2011). No patients in this study underwent surgical lung biopsy to diagnose IPF.

According to the sequential reading method, a board-certificated pulmonologist (N.K.) and a board-certificated radiologist (M.M.) independently reviewed all subjects’ CT scans and classified them into four categories: UIP pattern, possible UIP pattern, inconsistent with UIP pattern, and without ILD. Accordance of their classifications was considered a definite diagnosis. When their classifications differed, two other board-certificated pulmonologists (T.T. and A.T.) carefully reviewed the scans independently; the diagnosis was considered definite if the classifications of three of the four readers were in accord. When two readers agreed on one classification and the other two another, the case was discussed until all four readers agreed on a diagnosis (this procedure was required in two cases).

Diagnoses of AEs of ILD were made in accordance with criteria detailed in previous studies as follows: (1) worsening of dyspnea within 30 days; (2) new radiologic bilateral ground-glass abnormality and/or consolidation superimposed on a background of reticular shadows or honeycombing; (3) no evidence of pulmonary infection; and (4) exclusion of alternative causes, including left heart failure, pulmonary embolism, and acute lung injury of identifiable cause (Collard et al. 2007; Kinoshita et al. 2012).

### Statistical analysis

PFS was defined as the time between the start of chemotherapy or molecular targeted therapy and diagnosis of disease progression or death. OS was defined as the time between the date of diagnosis and date of death from any cause. PFS and OS curves were constructed by the Kaplan–Meier method, and differences in survivals compared using the log-rank test. Fisher’s exact test and Student’s *t* test were used to analyze patient characteristics and the significance of the association of AE with ILD or IPF. Laboratory and pulmonary function data are presented as mean ± SD. All statistical analyses were performed using Ekuseru-Toukei 2015 (Social Survey Research Information, Tokyo, Japan).

## Results

### Patient selection

In all, 285 patients with pathologically confirmed advanced (stage IIIB or IV) NSCLC were identified for this study, 29 of whom had received definitive thoracic irradiation, four had another concomitant active malignancy (malignant lymphoma, renal, gastric, or colon cancer), and 34 had received only best supportive care. Thus, 218 patients were included in this study. Samples were obtained by transbronchial biopsy (131 cases), percutaneous biopsy (71 cases including 31 pleural effusions), surgical resection (9 cases), and others such as biopsy at different departments (7 cases).

### Classification of ILD and diagnosis of IPF

Relevant characteristics of patients treated with chemotherapy and/or molecular targeted therapy are shown in Table 1. ILD was identified in 53/218 patients (24.3 %): 35 were diagnosed as having UIP, 15 possible UIP, and three inconsistent with UIP. One patient had dermatomyositis-related UIP pattern, and the remaining 34 with UIP pattern were diagnosed as having IPF (15.6 % of 218 patients).

**Table 1:** Characteristics of patients treated with chemotherapy and/or molecular targeted therapy

	Non-ILD (*n* = 165)	ILD			
		All ILD (*n* = 53)	*P* (vs. non-ILD)	IPF (*n* = 34)	*P* (vs. non-ILD)
Age, years (range)	66 (30–91)	71 (57–86)	0.00027	70 (57–86)	0.0053
Sex					
Male	95 (58 %)	51 (96 %)	<0.0001	34 (100 %)	<0.0001
Female	70 (42 %)	2 (4 %)		0 (0 %)	
Smoking status					
Never	56 (34 %)	2 (4 %)	<0.0001	0 (0 %)	<0.0001
Ever	109 (66 %)	51 (96 %)		34 (100 %)	
Pack-year, average	52	34		67	
PS					
0–1	134 (81 %)	43 (81 %)	1.0000	27 (79 %)	0.8123
2–4	31 (19 %)	10 (19 %)		7 (21 %)	
Histology					
Adeno	129 (78 %)	21 (40 %)	<0.0001	12 (35 %)	<0.0001
Squamous	22 (13 %)	19 (36 %)	0.0005	12 (35 %)	0.0046
Adenosquamous	1	2		2	
Large	1	2		1	
Non-small	12	9		7	
Stage					
IIIB	14	6	0.5854	3	1.0000
IV	151	47		31	
EGFR mutation					
Yes	53 (32 %)	1 (2 %)	<0.0001	0 (0 %)	<0.0001
No	112 (68 %)	52 (98 %)		34 (100 %)	

*ILD* interstitial lung disease, *IPF* idiopathic pulmonary fibrosis, *PS* performance status, *EGFR* epidermal growth factor receptor

Patients with ILD were significantly older than those without ILD and more often male and smokers (Table 1). The frequency of adenocarcinoma was lower, and that of squamous cell carcinoma was higher in patients with ILD than in those without it (40 vs. 78 % for adenocarcinoma, and 36 and 13 % for squamous cell carcinoma, respectively). *EGFR* mutation was detected in 53/112 non-ILD patients (32 %) and in only one of 53 ILD patients (2 %). These differences were more extreme in patients with IPF, all of whom were male smokers. Adenocarcinoma and squamous cell carcinoma histology each comprised 35 % of cases, and no *EGFR* mutations were detected. Average of percent vital capacity (%VC) is significantly lower in IPF than in non-ILD patients (76.4 % vs 89.1 %, respectively, *P* = 0.0115, data not shown).

### Responses to chemotherapy and/or molecular targeted therapy

First-line therapy was cytotoxic chemotherapy in 179/218 patients and EGFR-TKI in the remaining 39 (Table 2). Fewer than three cycles of first-line chemotherapy were received by 72 and 82 % of ILD and IPF patients, respectively, both percentages being significantly higher than for non-ILD patients (37 %, *P* < 0.0001). RR evaluated by RECIST was 55, 37, and 31 % for non-ILD, ILD, and IPF patients, respectively. Disease control rate (DCR) was 87, 71, and 53 % in non-ILD, ILD, and IPF patients, respectively. RR and DCR were significantly lower in ILD and IPF patients than in non-ILD patients. When patients with *EGFR* mutation were excluded (*n* = 54), RR was 44, 35, and 31 % for non-ILD, ILD, and IPF patients, respectively. DCR was 85, 67, and 53 % for non-ILD, ILD, and IPF patients, respectively. Although RR was not significantly different in subjects with *EGFR* wild type (WT), the DCR was significantly lower in ILD and IPF than in non-ILD patients.

**Table 2:** Response to first-line chemotherapy/molecular targeted therapy

	Non-ILD (*n* = 165)	ILD			
		All ILD (*n* = 53)	*P* (vs. non-ILD)	IPF (*n* = 34)	*P* (vs. non-ILD)
Number of cycles of chemotherapy, average (range)	5 (1–24)	4 (1–34)	0.1121	3 (1–12)	0.0014
Number of patients who received fewer than three cycles (%)	61 (37 %)	38 (72 %)	<0.0001	28 (82 %)	<0.0001
All patients					
CR	4	0		0	
PR	86	19		10	
SD	51	17		7	
PD	22	15		15	
NA	2	2		2	
Response rate	55 %	37 %	0.0363	31 %	0.0193
Disease control rate	87 %	71 %	0.0180	53 %	0.0001
Patients without EGFR mutation					
CR	2	0		0	
PR	46	18		10	
SD	46	17		7	
PD	16	15		15	
NA	2	2		2	
Response rate	44 %	35 %	0.3910	31 %	0.2278
Disease control rate	85 %	67 %	0.0304	53 %	0.0004

*ILD* interstitial lung disease, *IPF* idiopathic pulmonary fibrosis, *CR* complete response, *PR* partial response, *SD* stable disease, *PD* progressive disease, *NA* not assessed

### Shorter PFS and OS in patients with ILD or IPF

**Fig. 1:**
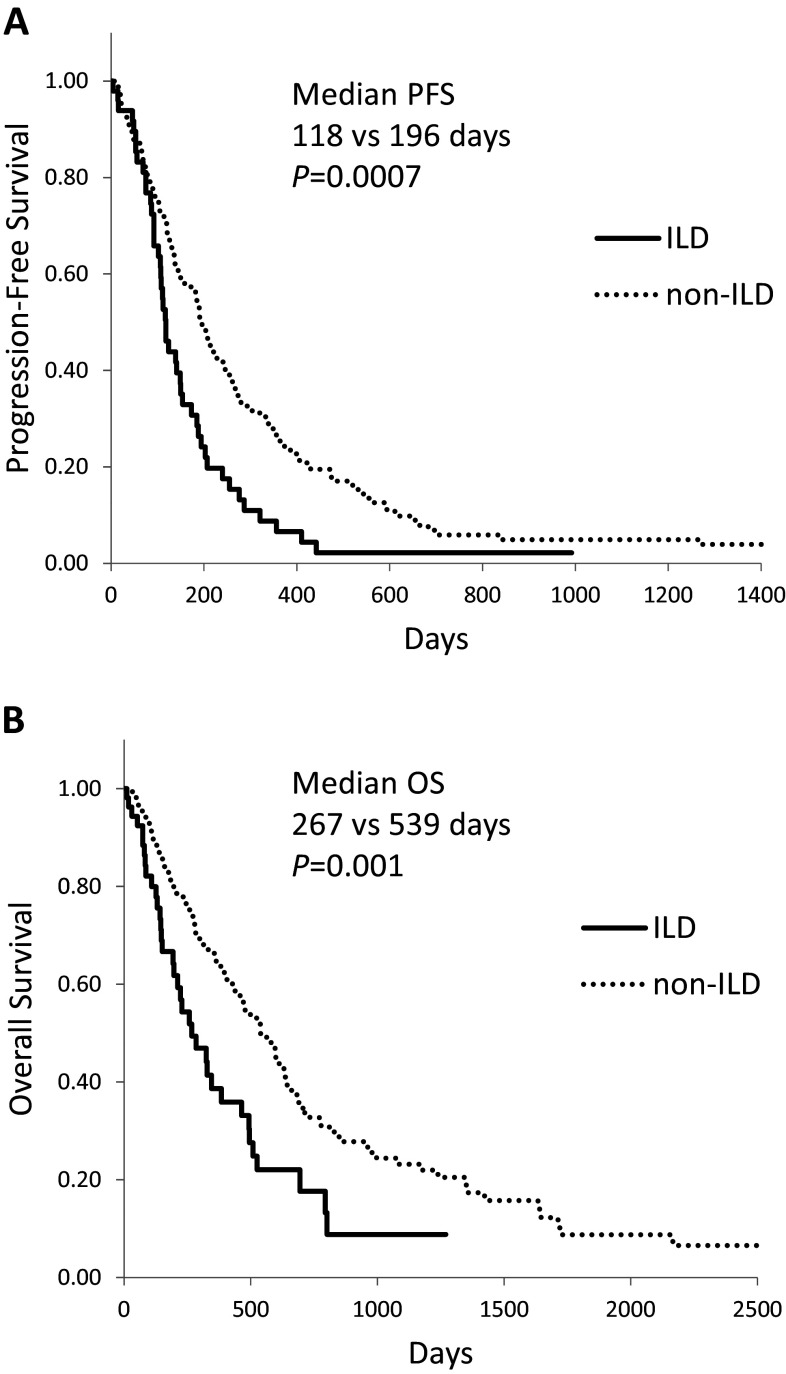
Kaplan–Meier curves of **(a)** progression-free survival and **(b)** overall survival in advanced NSCLC patients with interstitial lung disease (ILD) who received chemotherapy and/or molecular targeted therapy

**Fig. 2:**
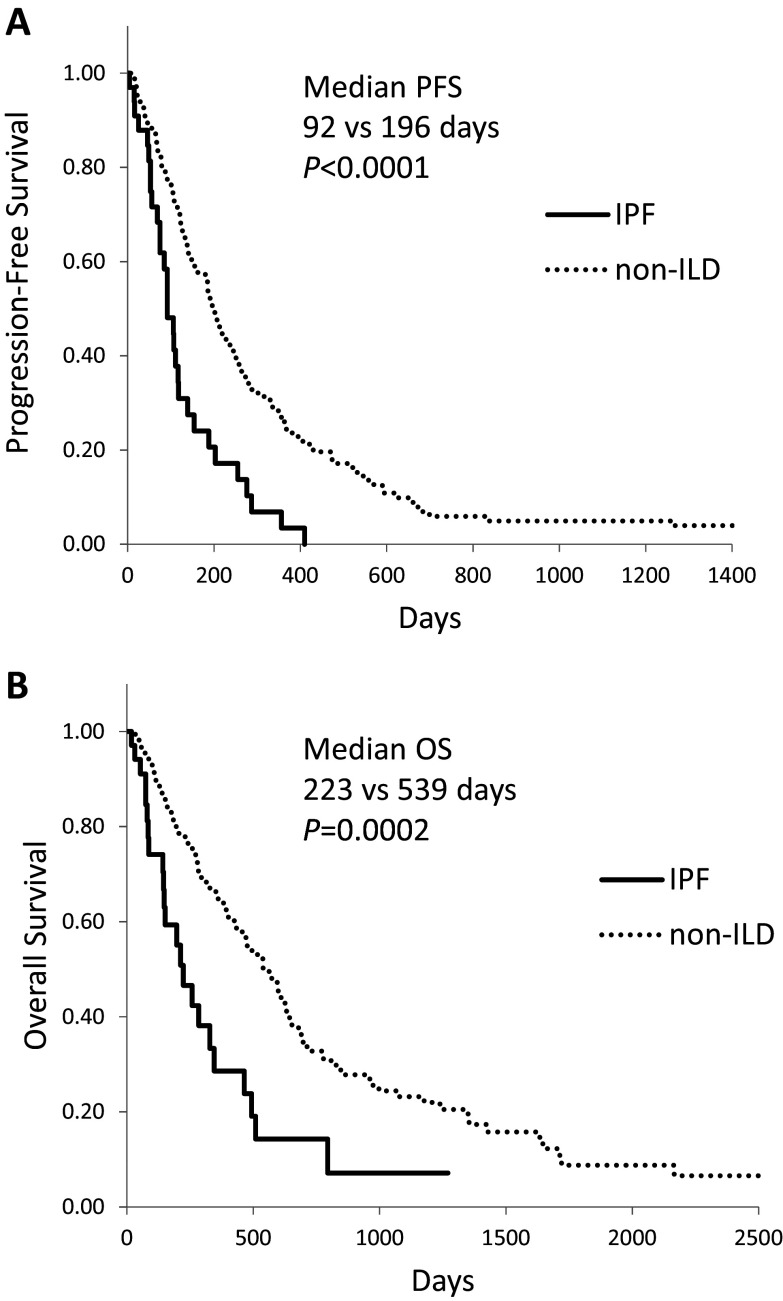
Kaplan–Meier curves of **(a)** progression-free survival and **(b)** overall survival in advanced NSCLC patients with idiopathic pulmonary fibrosis (IPF) who received chemotherapy and/or molecular targeted therapy

Kaplan–Meier survival curves for NSCLC patients who received chemotherapy and/or molecular targeted therapy showed a significantly shorter median PFS (Fig. 1a, 118 vs. 196 days, *P* = 0.0007) and OS (Fig. 1b, 267 vs 539 days, *P* = 0.001) in ILD than in non-ILD patients. In patients with IPF, median PFS (92 days, *P* < 0.0001) and OS (223 days, *P* = 0.0002) were even shorter (Fig. 2).

Univariate analysis by the log-rank test identified that male sex, smoking history, poor performance status (PS), non-adenocarcinoma histology, and *EGFR*-WT were associated with poor PFS and/or OS (Table 3). Multivariate analysis by Cox proportional hazards model showed that poor PS, absence of *EGFR* mutation, and presence of IPF were identified as poor prognostic factors for PFS and OS.

**Table 3:** Risk factors associated with PFS and OS

	*n*	PFS				OS			
		Median (days)	Univariate analysis	Multivariate analysis		Median (days)	Univariate analysis	Multivariate analysis	
			*P* value	HR (95 % CI)	*P* value		*P* value	HR (95 % CI)	*P* value
Age									
Older (≥75 years)	56	150	0.0827	1.33 (0.94–1.88)	0.1082	314	0.0145	1.46 (1.00–2.15)	0.0528
Younger (<75 years)	162	186				539			
Sex									
Female	72	228	0.0403	1.05 (0.67–1.67)	0.8247	638	0.0030	0.81 (0.48–1.36)	0.4219
Male	146	149				398			
Smoking status									
Never	58	237	0.0405	0.84 (0.52–1.33)	0.4536	610	0.0242	0.95 (0.57–1.61)	0.8595
Ever	160	149				430			
PS									
0–1	178	202	<0.0001	0.35 (0.24–0.52)	<0.0001	538	<0.0001	0.24 (0.16–0.36)	<0.0001
2–4	40	79				139			
Histology									
Adeno	150	190	0.0430	1.02 (0.71–1.47)	0.8954	539	0.0009	1.00 (0.67–1.49)	0.9929
Others	68	150				283			
Stage									
IIIB	20	185	0.7203	0.78 (0.47–1.28)	0.3246	495	0.9407	0.90 (0.52–1.56)	0.7136
IV	198	180				471			
EGFR mutation									
Yes	54	304	0.0003	0.55 (0.37–0.82)	0.0037	771	0.0001	0.51 (0.33–0.81)	0.0042
No	164	149				360			
ILD									
IPF	34	92	0.00002	1.98 (1.26–3.11)	0.0030	223	0.0002	1.74 (1.03–2.92)	0.0373
Non-ILD	165	196				539			

*PFS* progression-free survival, *OS* overall survival, *HR* hazard ratio, *PS* performance status, *EGFR* epidermal growth factor receptor, *ILD* interstitial lung disease, *IPF* idiopathic pulmonary fibrosis

**Table 4:** Associations between chemotherapeutic regimens and acute exacerbation of ILD and IPF

	ILD					IPF				
	*n*	AE (−)	AE (+)	Incidence (%)	*P* value	*n*	AE (−)	AE (+)	Incidence (%)	*P* value
Docetaxel										
Containing	38	31	7	18.4	0.0472	24	19	5	20.8	0.0802
Not containing	58	55	3	5.2		29	28	1	3.4	
Pemetrexed										
Containing	15	13	2	13.3	0.6531	8	7	1	12.5	1.0000
Not containing	81	73	8	9.9		45	40	5	11.1	
Paclitaxel/nab-paclitaxel										
Containing	12	12	0	0.0	0.3533	5	5	0	0.0	1.0000
Not containing	84	74	10	11.9		48	42	6	12.5	

*ILD* interstitial lung disease, *IPF* idiopathic pulmonary fibrosis, *AE* acute exacerbation

Because *EGFR* mutation status is associated with survival and differs between non-ILD and ILD/IPF patients, PFS and OS were assessed in these patients. Interestingly, PFS and OS were still shorter in ILD and IPF patients with *EGFR*-WT. Median PFS was 180, 118, and 92 days for non-ILD, ILD, and IPF patients, respectively (*P* = 0.0301 for non-ILD vs. ILD and *P* = 0.0018 for non-ILD vs. IPF). Median OS was 458, 258, and 223 days for non-ILD, ILD, and IPF patients, respectively (*P* = 0.0202 for non-ILD vs. ILD and *P* = 0.0126 for non-ILD vs. IPF).

### Factors associated with AEs of ILD

AEs were observed in 10/53 patients (18.9 %) with ILD (Table 5). Limited to patients with IPF, AEs developed in 6/34 patients (17.6 %). No factors associated with the occurrence of AE were identified. However, the incidence of AEs differed between chemotherapeutic regimens (Table 4). The number of regimen types received by each patient was counted as shown by the following example: carboplatin plus pemetrexed plus bevacizumab followed by pemetrexed plus bevacizumab was classified as one regimen type. The same regimen administered to two patients was counted as two regimens. In all, 96 regimens were administered to 53 patients with ILD. When they contained docetaxel, the incidence of AEs was 18.4 % (7/38 regimens), which is significantly higher than for regimens without docetaxel (5.2 %, *P* = 0.0472). In IPF patients, docetaxel-containing regimens resulted in a trend toward higher incidence of AEs (5/24 patients; 20.8 %), whereas only 1/29 regimens (3.4 %) without docetaxel led to AEs (*P* = 0.0802); this difference is not statistically significant. No ILD patients received EGFR-TKI.

**Table 5:** Patient characteristics associated with acute exacerbations of ILD and IPF

	ILD			IPF		
	AE (−) (*n* = 43)	AE (+) (*n* = 10)	*P* value	AE (−) (*n* = 28)	AE (+) (*n* = 6)	*P* value
Age						
Older (≥75 years)	13	5	0.2792	9	3	0.6410
Younger (<75 years)	30	5		19	3	
Sex						
Female	1	1	0.3447	0	0	1.0000
Male	42	9		28	6	
Smoking status						
Never	1	1	0.3447	0	0	1.0000
Ever	42	9		28	6	
PS						
0–1	36	7	0.3761	23	4	0.5798
2–4	7	3		5	2	
Histology						
Adeno	19	5	0.7411	10	2	1.0000
Others	24	5		18	4	
Stage						
IIIB	5	1	1.0000	3	0	1.0000
IV	38	9		25	6	
EGFR mutation						
Yes	0	1	0.1887	0	0	1.0000
No	43	9		28	6	
LDH	283 ± 127	314 ± 126	0.5208	277 ± 114	340 ± 143	0.3829
KL-6	808 ± 608	688 ± 372	0.5435	865 ± 664	807 ± 460	0.8625
%VC	81.8 ± 19.8	84.4 ± 18.5	0.7426	78.0 ± 18.0	71.6 ± 13.8	0.4691
%FVC	79.7 ± 20.2	80.6 ± 18.7	0.9085	78.1 ± 18.3	67.2 ± 13.8	0.2263

*ILD* interstitial lung disease, *IPF* idiopathic pulmonary fibrosis, *AE* acute exacerbation, *PS* performance status, *EGFR* epidermal growth factor receptor, *LDH* lactate dehydrogenase, *VC* vital capacity, *FVC* forced vital capacity

Median PFS in patients with and without AE was 118 versus 124 days (*P* = 0.4244), and median OS was 142 versus 284 days (*P* = 0.1099). Although these differences are not statistically significant, 6/10 patients died quickly after the onset of their AEs (Table 6). All patients who developed AEs were treated with corticosteroids, including methylprednisolone pulse therapy, and received no further chemotherapy.

**Table 6:** Outcomes of ten patients with acute exacerbations of ILD

No.	Classification of ILD	Associated regimens (chemotherapeutic line)	Cycles received	Onset of AE (days from chemotherapy)	Outcome of AE	Survival (days from onset of AE)
1	Possible UIP	Pemetrexed (second)	1	13	Died	16
2	Inconsistent with UIP	S-1 (third)	1	26	Died	2
3	Possible UIP	Carboplatin and docetaxel (first)	5	150	Died	36
4	IPF	Carboplatin and docetaxel (first)	1	15	Died	12
5	IPF	Carboplatin and pemetrexed (first)	2	41	Recovered	81
6	IPF	Carboplatin and docetaxel (first)	3	22	Died	4
7	IPF	Docetaxel (first)	2	52	Recovered	80
8	IPF	Docetaxel (first)	2	46	Died	4
9	Possible UIP	Docetaxel (second)	2	28	Recovered	385
10	IPF	Docetaxel (first)	1	18	Recovered	108

*ILD* interstitial lung disease, *UIP* usual interstitial pneumonia, *IPF* idiopathic pulmonary fibrosis, *AE* acute exacerbation

The reasons for discontinuation of first-line chemotherapy within three cycles were investigated (Table 7). Interestingly, discontinuation because of adverse events occurred significantly more often in ILD and IPF than in non-ILD patients (42, 46, and 21 %, respectively). Importantly, 6/16 adverse events that resulted in discontinuation of chemotherapy were AEs in ILD patients. In IPF patients, AEs comprise 6/13 adverse events.

**Table 7:** Reasons for discontinuation of first-line chemotherapy within three cycles

	Non-ILD (*n* = 61)	ILD			
		All ILD (*n* = 38)	*P* (vs. non-ILD)	IPF (*n* = 28)	*P* (vs. non-ILD)
PD	21 (34 %)	14 (37 %)	0.8316	13 (46 %)	0.3490
Deterioration in PS	12 (20 %)	7 (18 %)	1.0000	5 (18 %)	1.0000
Adverse events	13 (21 %)	16 (42 %)	0.0403	13 (46 %)	0.0234
Acute exacerbation	–	6		6	
Others	16 (26 %)	4 (11 %)	0.0735	1 (4 %)	0.0100

*Note* Because numbers are shown as events, the total number may exceed patient numbers and the total percentage will exceed 100 %

*ILD* interstitial lung disease, *IPF* idiopathic pulmonary fibrosis, *PD* progressive disease, *PS* performance status

## Discussion

In the present study, we demonstrated that (1) characteristics of ILD/IPF patients differed significantly from those of non-ILD patients; in particular, no IPF patients had EGFR mutation-positive tumors; (2) the presence of either IPF or ILD was associated with shorter PFS and OS in patients with advanced NSCLC who received chemotherapy; (3) DCR was significantly lower in ILD and IPF patients than in non-ILD patients, even those with *EGFR*-WT; and (4) AEs of ILD occurred more frequently after docetaxel-containing regimens than after other regimens.

The present study clearly demonstrated several differences in the characteristics of non-ILD and ILD/IPF patients. Most ILD and all 34 IPF patients were male smokers and had *EGFR*-WT. It is particularly interesting that no IPF patients had *EGFR* mutation-positive cancers, which has been shown for the first time although a previous study has reported a correlation between preexisting ILD and *EGFR*-WT adenocarcinoma (Fujimoto et al. 2013). In that study, only one of 31 ILD patients with lung adenocarcinoma had an *EGFR* mutation; this patient had a non-UIP radiographic pattern (Fujimoto et al. 2013). The current study extended this finding in that our one ILD patient with *EGFR* mutation also had a radiologic pattern inconsistent with UIP pattern. The frequency of squamous cell carcinoma was higher in IPF than in non-ILD patients (35 vs. 13 %). In ILD patients, most tumors reportedly develop in the area affected by ILD (Fujimoto et al. 2013; Kanaji et al. 2015). The current study provides evidence that carcinogenesis in IPF differs from that in non-ILD patients in that it is not associated with *EGFR* mutation.

Consistent with previous studies (Borchers et al. 2011; Watanabe et al. 2013), IPF was the commonest type of ILD (34/53 patients; 64 %). Even in patients with *EGFR*-WT, PFS and OS were significantly shorter in IPF than in non-ILD patients. These findings indicate that the presence of IPF predicts an extremely poor prognosis in patients with advanced NSCLC. Lower %VC (<80 %) was also associated with shorter survivals (120 vs 196 days for PFS, *P* = 0.0043, and 266 vs 538 days for OS, *P* = 0.0023, respectively, data not shown). Shorter survival in lower %VC has been reported in patients with NSCLC and ILD who underwent surgical resection (Sato et al. 2015).

Another important finding of the current study is regarding response to chemotherapy/molecular targeted therapy. RR and DCR in ILD/IPF patients were lower than that in non-ILD patients. *EGFR* mutation was not identified in any of the 34 IPF patients, whereas 32 % of non-ILD patients harbored *EGFR* mutations. Because the response to EGFR-TKI is much better in tumors with *EGFR* mutation than in *EGFR*-WT, the lower RR in IPF patients may be associated with the absence of *EGFR* mutations. Indeed, the difference in RR was not significant for *EGFR*-WT patients; interestingly, however, a difference in DCR was observed, even in patients without *EGFR* mutations.

There are several possible mechanisms that could explain the lower DCR and shorter survival of ILD/IPF patients. First, ILD/IPF patients may experience adverse events more frequently. Indeed, adverse events, including AEs, and PD were the main reasons for discontinuing first-line therapy within three cycles in patients with ILD/IPF, suggesting that AEs of ILD/IPF may have made an important contribution to the lower DCR and shorter survival. Consistent with this, coexisting ILD is reportedly associated with a high risk of developing chemotherapy-induced ILD (Sakurada et al. 2015). Chemotherapeutic strategies that do not induce AEs or deterioration of ILD are needed.

Second, an altered drug delivery system may lead to the lower response and shorter survival of ILD/IPF patients. Histological features of the UIP pattern include marked pulmonary fibrosis and architectural distortion (Raghu et al. 2011). Alveolar epithelial cell damage, dysregulation of fibroblasts, and vascular injury and aberrant angiogenesis related to vascular remodeling are key elements of lung fibrosis (Selman and Pardo 2006; Wémeau-Stervinou et al. 2012). Destruction of normal vasculature would logically reduce the local delivery of chemotherapeutic agents to a tumor.

Third, acquired drug resistance may result in lower DCR and shorter survival in ILD/IPF patients. In this regard, transforming growth factor (TGF)-*β* may have a role. Many cytokines and growth factors, including TGF-*β*, are reportedly associated with the development of ILD/IPF (Das et al. 2014; Kanaji et al. 2014). TGF-*β* concentrations are higher in bronchoalveolar lavage fluid obtained from IPF than from control patients (Khalil et al. 2001). TGF-*β* is reportedly associated with chemoresistance in colon cancer (Li et al. 2015). Additionally, TGF-*β* is a major regulator of epithelial–mesenchymal transition in the alveolar epithelia (Kasai et al. 2005; Miyazono 2009). In epithelial–mesenchymal transition, cancer cells seemingly acquire chemoresistance in lung and other types of cancers (Jiang et al. 2015; Li et al. 2014; Wang et al. 2015). Thus, increased TGF-*β* in the lung’s microenvironment may be a causative factor for lower DCR and shorter survival in NSCLC patients with ILD/IPF.

Regarding AEs of ILD, several studies have reported docetaxel-induced AE (Tamiya et al. 2012; Watanabe et al. 2015). In 35 patients with NSCLC and IP treated with docetaxel monotherapy, the incidence of AEs of IP was 14.3 % (5/35 patients). Three of the five patients who developed AEs of IP died (Watanabe et al. 2015). In another study, deterioration of ILD was observed in 7/27 patients (25.9 %) with NSCLC and preexisting ILD (Tamiya et al. 2012). In contrast, 6/309 patients (1.9 %) without preexisting ILD reportedly developed it after receiving docetaxel (Tamiya et al. 2012). In the current study, there was also a higher incidence of AEs of ILD/IPF after docetaxel-containing chemotherapy. Of note, AEs also occurred in 3/15 patients with radiographic possible UIP pattern (20 %). Several studies have reported that possible UIP pattern often corresponds with pathologically confirmed UIP (Sumikawa et al. 2014; Raghu et al. 2014). Radiographic possible UIP pattern should be considered clinically similar to UIP when choosing treatment strategies for patients with advanced NSCLC. Administration schedule also affects the incidence of AEs (higher incidence in a weekly than in triweekly administration) (Chen et al. 2006). Anti-microtubule activity by taxanes itself seems not to be related in the development of AE because the incidence of AE in paclitaxel is different from that in docetaxel. Thus, factors other than anticancer activity might be associated with docetaxel-related AE although an actual mechanism has never been reported.

No optimal chemotherapeutic regimen for patients with advanced NSCLC and underlying ILD has been established (Watanabe et al. 2015). A combination of carboplatin and weekly paclitaxel may be a treatment option for NSCLC with ILD (Minegishi et al. 2011). Eighteen patients with ILD, including six with IPF, treated with carboplatin and paclitaxel achieved PFS and median survival time of 5.3 and 10.6 months, respectively (Minegishi et al. 2011). Only one patient (5.6 %) with IPF developed an AE (after four cycles of chemotherapy). However, in another study 4/15 patients with NSCLC and ILD who received carboplatin and paclitaxel developed grade 3 or higher pneumonitis (27 %) (Shukuya et al. 2010); this high incidence cannot be ignored. In a phase III trial, administration of albumin-bound paclitaxel (nab-paclitaxel) as first-line treatment in patients with advanced NSCLC without ILD was effective and resulted in a higher RR than conventional solvent-based paclitaxel (Socinski et al. 2012). Currently, several clinical trials are assessing a combination of carboplatin and nab-paclitaxel for advanced NSCLC with ILD.

Nintedanib is a multiple tyrosine kinase inhibitor, and INPULSIS-2 trial demonstrated that nintedanib reduced the time to first AE compared with placebo (Richeldi et al. 2014). In addition, a combination of docetaxel and nintedanib showed a benefit on OS over docetaxel monotherapy as second-line treatment (Reck et al. 2014). The addition of nintedanib to cytotoxic agents such as docetaxel may be more effective with fewer adverse events.

The prevalence of ILD and IPF in this study was 24.3 and 15.6 %, respectively, and seems to be higher than in previous reports (7.3–14 %) (Kinoshita et al. 2012; Nishino et al. 2015). However, among 387 patients who received surgical resection, 65 (16.8 %) were confirmed as underlying IPF (Goto et al. 2014). Location of the institution would greatly influence the prevalence of ILD and IPF as well as smoking status.

The limitations of this study are as follows. First, this was a retrospective, single-center study; a prospective, multicenter large study including both non-ILD and ILD patients is desirable to further investigate clinical differences in response to chemotherapy. Second, all patients were Japanese: Some studies have shown ethnic differences in incidence of drug-induced lung injury and other pulmonary diseases (Azuma et al. 2008; Natsuizaka et al. 2014). The incidence of AEs of ILD/IPF may vary between different ethnicities.

## Conclusions

This study showed, for the first time, that patients with advanced NSCLC and IPF have shorter PFS and OS and lower DCR than patients without ILD. Docetaxel-containing chemotherapeutic regimens were more frequently associated with AEs than other regimens. Because survival after onset of AEs is usually short, more effective therapeutic strategies that do not induce AEs should be identified.
